# DHA and EPA Alleviate Epileptic Depression in PTZ-Treated Young Mice Model by Inhibiting Neuroinflammation through Regulating Microglial M2 Polarization and Improving Mitochondrial Metabolism

**DOI:** 10.3390/antiox12122079

**Published:** 2023-12-06

**Authors:** Yueqi Yang, Lu Chen, Ning Zhang, Yingcai Zhao, Hongxia Che, Yuming Wang, Tiantian Zhang, Min Wen

**Affiliations:** 1 Institute of Biopharmaceutical Research, Liaocheng University, Liaocheng 252059, China; qiqiyang77@163.com (Y.Y.); 17781028137@163.com (L.C.); zhangning@lcu.edu.cn (N.Z.); 2 College of Food Science and Engineering, Ocean University of China, Qingdao 266404, China; zhaoyingcai@stu.ouc.edu.cn (Y.Z.); wangyuming@ouc.edu.cn (Y.W.); zhangtiantian@ouc.edu.cn (T.Z.); 3 College of Marine Science and Biological Engineering, Qingdao University of Science and Technology, Qingdao 266042, China; 03474@qust.edu.cn; 4 Pet Nutrition Research and Development Center Gambol Pet Group Co., Ltd., Liaocheng 252000, China

**Keywords:** DHA, EPA, neuroinflammation, oxidative stress, epileptic depression, young mice

## Abstract

Depression is the most common complication of childhood epilepsy, leading to a poor prognosis for seizure control and poor quality of life. However, the molecular mechanisms underlying epileptic depression have not been completely elucidated. Increasing evidence suggests that oxidative stress and neuroinflammation are major contributors to depression. The positive effects of dietary supplementation with docosahexaenoic acid (DHA) and eicosapentaenoic acid (EPA) on depression have been previously reported. However, knowledge regarding the effects of EPA and DHA in managing depressive symptoms in pediatric patients with epilepsy is limited. Therefore, this study aims to investigate the effects of EPA and DHA on epileptic depression in a pentylenetetrazole (PTZ)-treated young mouse model. Three-week-old mice were fed a DHA- or EPA-enriched diet for 21 days and treated with PTZ (35 mg/kg, i.p.) every other day for a total of 10 times. EPA was more effective than DHA at alleviating PTZ-induced depressive symptoms. Pathological results revealed that DHA and EPA significantly improved neuronal degeneration in the hippocampus. Analysis of the mechanism revealed that DHA and EPA mitigated PTZ-induced myelin damage by increasing the protein levels of CNPase, Olig2, and MBP. Furthermore, both DHA and EPA reduced neuroinflammation by promoting microglial M2 polarization and suppressing the LCN2-NLRP3 inflammasome pathway. Notably, EPA polarized microglia towards the M2 phenotype. In addition, DHA and EPA decreased oxidative stress by inhibiting NOX2 and enhancing mitochondrial metabolism through the increased expression of mitochondrial respiratory chain complex I-V proteins. These findings suggest that DHA and EPA can be used as effective interventions to improve depression in children with epilepsy, with EPA being a particularly favorable option.

## 1. Introduction

Epilepsy, a neurological disorder prevalent in children, is characterized by recurrent epileptic seizures. Meanwhile, comorbid conditions, such as cognitive, behavioral, and psychiatric disorders also frequently occur with childhood epilepsy [1]. Depression is the most frequent psychiatric comorbidity of epilepsy, affecting up to one-third of children with epilepsy, but remains underrecognized and undertreated [2,3]. It is worth noting that there exists a bidirectional relationship between childhood epilepsy and depression, whereby depression increases the risk of epilepsy and vice versa [4,5]. Furthermore, depression can significantly impact quality of life and may even worsen the prognosis for seizure management [2,6,7].

Mounting evidence suggests that neuroinflammation plays a critical role in depression [8] and contributes to the onset of epileptic seizures and epileptogenesis [9,10]. Recurrent epileptic seizures may lead to the development of chronic inflammation, which further contributes to depression [11]. Microglia, the main innate immune cells in the central nervous system, are rapidly activated in response to tissue repair and host defense against infectious stimuli, and exhibit two major phenotypes: M1 (pro-inflammatory) and M2 (anti-inflammatory) [12]. M1 polarization is characterized by the secretion of proinflammatory cytokines and an increased expression of iNOS. The M1 microglial phenotype has been suggested as a cause or consequence of epilepsy [10,13,14] and is conducive to the onset of depression [8]. Polarization of microglia into the M2 phenotype, characterized by a higher expression of arginase-1 (Arg1), facilitates the release of various anti-inflammatory factors to enhance neuronal protection [15]. Inhibition of microglial M1 polarization and/or induction of M2 polarization has been proposed as an effective therapeutic strategy for epilepsy [16] and depression [17,18]. However, the exact role and mechanism of microglial polarization in children with epilepsy have not yet been clarified.

Oxidative stress is the primary etiology of inflammatory disruptions [19]. Reactive oxygen species (ROS) and reactive nitrogen species (RNS) cause oxidative stress when antioxidant defenses fail. Interestingly, the brain, an organ with high oxygen consumption, is more susceptible to oxidative stress owing to its high metabolic rate and low antioxidant levels [20,21]. Recent evidence has highlighted the role of oxidative stress in brain disorders, such as Alzheimer’s disease [22], epileptogenesis [23], and depression [21,24]. Mitochondria are the primary site of ROS production, and their role has gained increased recognition in the progression of depression [25] and epileptogenesis. Additionally, NADPH oxidase (NOX) functions as a ubiquitous intracellular source of superoxide radicals through the enzymatic reduction of molecular oxygen and the subsequent oxidation of NADPH. The central nervous system contains cells that express NOX2, such as microglia and neurons. Moreover, NOX2 is upregulated in epilepsy [26] and depression [27]. NOX2-derived ROS are involved in neuroinflammation [28]. However, mechanisms underlying oxidative stress in childhood epilepsy remain unclear.

The primary treatment of epilepsy in children relies on AEDs. However, it should be noted that some AEDs may have adverse side effects and potentially worsen or trigger depression or anxiety [5]. There is growing interest in alternative and nutritional therapies for children epilepsy and depression [29,30]. Fish oil is widely recognized for its neuroprotective properties due to its rich eicosapentaenoic acid (EPA) and docosahexaenoic acid (DHA) contents, which promote neurodevelopment. Furthermore, an increasing body of evidence has demonstrated its efficacy in treating epilepsy and alleviating depression [31,32]. Notably, there is a discrepancy between EPA and DHA in terms of their effectiveness in improving depression [33] and epilepsy [32,34]. In addition, our previous study found that DHA and EPA prevented seizure and depression-like symptoms by inhibiting neuroinflammation via different modes-of-action in a PTZ- kindling model in adult mice [35]. However, the effects of EPA and DHA on children with epileptic depression remain unclear. Therefore, this study aimed to investigate the effects of EPA and DHA on epileptic depression in young mice.

## 2. Materials and Methods

### 2.1. Subject Animals and Experimental Design

The administration of PTZ can elicit convulsions that resemble absence seizures in humans, making it a valuable tool for establishing rodent models of epilepsy [36]. Forty male ICR mice (3 weeks old) were assigned to four groups (*n* = 10 per group): control (Con), PTZ kindling (PTZ), PTZ kindling + EPA (EPA), and PTZ kindling + DHA (DHA). Groups that received either EPA or DHA were fed diets enriched with 1% ethyl ester [23]. The compositions of the ingredients are provided in Supplemental Table S1.

### 2.2. The PTZ-Kindled Epilepsy Model Mice Construction

Pentylenetetrazole, the most commonly used GABAA receptor antagonist to induce epileptic seizures [37], was purchased from Sigma-Aldrich Chemical Co. Ltd. (Tokyo, Japan). A single intraperitoneal injection of 35 mg/kg PTZ was administered to the mice, followed by additional injections every other day, for a total of 11 doses. The Con group received saline injections [35]. Behavioral changes induced by PTZ were observed and recorded at 30 min post-administration. The number of seizures, seizure stage, and onset latency were objectively evaluated using the Racine scoring system. Seizure scores were recorded according to a previous study [38] with slight modifications, as follows: stage 1 (myoclonic jerk or straub tail), stage 2 (clonic seizure without loss of righting reflex), stage 3 (clonic seizures with loss of righting reflex), stage 4 (clonic tonic seizures), and stage 5 (death).

### 2.3. Behavioral Assays

Behavioral tests were conducted from 1:00 PM to 5:00 PM during the dark period of the circadian cycle.

#### 2.3.1. Tail Suspension Test (TST)

For the TST, the mice were suspended by their tails 50 cm above the ground and monitored for 5 min. Immobility time was recorded during the final 4 min.

#### 2.3.2. Forced Swimming Test (FST)

Individual mice were placed in transparent glass cylinders filled with water (40 cm in height, 15 cm in diameter, and 20 cm in water depth) maintained at a temperature of 25 °C. The behavior of the mice was recorded for 5 min using ANY-MAZE software version 6.3 (Stoelting Co., Wood Dale, IL, USA). During this period, both immobility time and immobility latency were measured.

#### 2.3.3. Open Field Test (OFT)

Nine squares were divided into 45 × 45 × 30 cm rectangular chambers for the open-field test. The mice were gently introduced into the center of the testing chamber for a 5-min recording period, and ANY-MAZE software version 6.3 (Stoelting Co., Wood Dale, IL, USA) was used to analyze the number and duration of entries into the center.

### 2.4. Western Blot and ELISA Analysis

As in our previous study [22], we lysed the tissue in modified RIPA buffer to obtain the required proteins. The primary antibodies of Nox2 (ab310337, 1:1000 dilution), NDUFB8 (ab192878, 1:5000 dilution), SDHB (ab175225, 1:50,000 dilution), UQCRC2 (ab203832, 1:2000 dilution), MTCO1 (ab203912, 1:1000 dilution), NLRP3 (ab263899, 1:1000 dilution), LCN2 (ab216462, 1:1000 dilution), Trem2 (ab305103, 1:1000 dilution), IL-1β (ab283818, 1:1000 dilution), IBA1 (ab178846, 1:1000 dilution), Olig2 (ab109186, 1:2000 dilution), NeuN (ab177487 1:1000 dilution) and INOS (ab178945, 1:1000 dilution) were purchased from Abcam (Cambridge, MA, USA). The primary antibodies of MBP (bs-0380R, 1:1000 dilution), CNPase (bs-1000R, 1:1000 dilution), and ASC (bs-41334R, 1:1000 dilution) were purchased from Bioss (Bejing, China). The primary antibodies of Caspase-1 (A0964, 1:2000 dilution), Arg1 (A4923, 1:5000 dilution) and ATP5A1 (A11217, 1:1000 dilution) were purchased from ABclonal (Wuhan, China). The primary antibodies of β-actin (66009-1-Ig) were used at a 1:2000 dilution, and the secondary antibodies included HRP-conjugated Affinipure Goat Anti-Mouse IgG (H + L) (Cat No. SA00001-1) and horseradish peroxidase-conjugated AffiniPure goat anti-rabbit IgG (H + L) (Cat No. SA00001-2), all purchased from Proteintech (Chicago, IL, USA) and used at a dilution of 1:5000. ECL Western blotting substrate was used for the development of blots, and a UVP Auto Chemi Image system (Tanon 4600SF, Shanghai, China) was employed to visualize luminescence.

The level of MDA (Nanjing Jiancheng Bioengineering Institute, A003-1-2, Nanjing, China) and GSH (Nanjing Jiancheng Bioengineering Institute, A006-2-1, Nanjing, China), and the activity of SOD (Nanjing Jiancheng Bioengineering Institute, A001-1-1, Nanjing, China) were analyzed using kits.

### 2.5. Nissl Staining

Nissl staining was performed using Nissl Staining Solution (Nissl Staining Solution (Cresyl Violet), G3410, Solarbio, Beijing, China) according to the manufacturer’s instructions. The paraffin-embedded brains were sectioned using a microtome. Sections were dewaxed, rehydrated, and immersed in a methylene blue staining solution for 10 min. After immersion in Nissl differentiation solution for 3 s, the sections were rinsed with water and subsequently dehydrated using pure alcohol. Sections were observed and recorded using a bright-field microscope.

### 2.6. Fluoro-Jade B (FJB) Staining

Fluoro-Jade B is a fluorescein derivative with anionic properties that specifically binds to denatured neurons, resulting in green fluorescence emission. Therefore, it can effectively label denatured necrotic neurons within the neural tissue [39]. Paraffin-embedded brain tissue sections were subsequently incubated in 0.06% potassium permanganate solution for 10 min to effectively suppress endogenous background signals. Next, the sections were immersed in a staining solution containing FJB (Sigma-Aldrich, Saint Louis, MO, USA) for 30 min. Subsequently, microphotographs were captured using a fluorescence microscope.

### 2.7. Neuronal Nuclei (NeuN) Staining

NeuN immunofluorescence staining was performed as described previously [40]. The mouse brain sections were subjected to NeuN staining (ab177487, 1:500), followed by incubation with a goat anti-rabbit IgG secondary antibody conjugated with alkaline phosphatase (ab6721, 1:500). Immunohistochemical staining was performed using the Vectastain^®^ ABC Kit (Vector Laboratories, Newark, CA, USA).

### 2.8. Immunofluorescence

Primary antibodies against IBA1 (ab178846, 1:500), and INOS (ab178945 1:500) were used, followed by species-specific secondary antibodies conjugated to Alexa Fluor 488 and 594. Digital images of whole stained slides were obtained by scanning with MIRAX MIDI digital whole slide scanners and analyzed using the Pannoramic Viewer software version 1.15.4 (Carl Zeiss MicroImaging, Jena, Germany) (3D Histech, Ltd., Ramsey, NJ, USA).

### 2.9. Statistical Analysis

Data are presented as means ± SEM. Multiple groups were compared using one-way analysis of variance (ANOVA) and Student’s *t*-test, with statistical significance defined as *p* < 0.05.

## 3. Results

### 3.1. Effects of EPA and DHA on PTZ-Induced Epileptic Seizure and Depressive-like Behaviors in Young Model Mice

**Figure 1:**
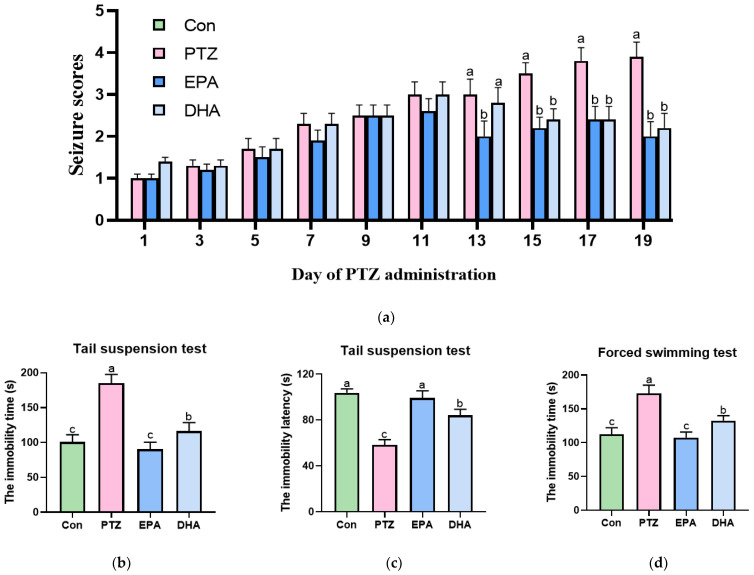

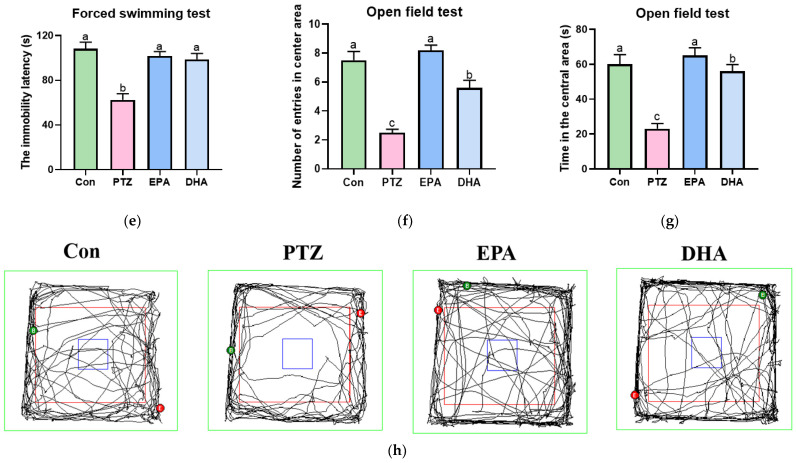
Effects of EPA and DHA on PTZ-induced epileptic seizure and depressive-like behaviors in young model mice. (**a**) The seizure scores in mice after PTZ treatment every other day; (**b**) the immobility time and (**c**) the immobility latency in the tail suspension test; (**d**) the immobility time and the (**e**) immobility latency in forced swimming test; (**f**) the number of entries in central area (the blue square) and (**g**) the time in central area in open field test; the green dots mean the start point and red dots mean the end point. (**h**) representative track plot data in probe trial in open field test. The data are presented as mean + SEM (*n* = 10); *p* < 0.05 was considered to be statistically significant. For quantification, a histogram was generated, where the group with the highest value is designated as a, followed by b–c. Groups with identical marker letters indicate no statistically significant differences, while those with different letters suggest significant differences between each group. Differences among groups were evaluated using one-way ANOVA, with significant differences indicated by different letters based on Student’s *t*-test.

Compared to the PTZ-treated mice, dietary DHA and EPA comparably alleviated seizure severity, reduced seizure numbers, and slowed seizure progression with prolonged latency (Figure 1a, *p* < 0.05). TST- and FST-treated mice displayed more immobility time than the Con group, indicating depressive and desperate emotions (*p* < 0.05). In contrast, the administration of EPA or DHA significantly reduced the immobility duration in both the TST and FST groups (Figure 1a–d, *p* < 0.05), with EPA showing a greater advantage. In addition, the PTZ-treated mice exhibited only a slight decrease in the number of entries and time spent in the central area compared to the Con group (Figure 1e,f). The administration of EPA and DHA notably increased the number of entries and the time spent in the central area, in which EPA was superior in terms of the number of entries (*p* < 0.05). A representative track of the four groups during the probe trial is shown in Figure 1g. These findings suggested that both EPA and DHA exerted an antidepressant effect in PTZ-treated young mice; specifically, EPA showed a better therapeutic effect.

### 3.2. Effects of EPA and DHA on Hippocampal Neuron Injury in PTZ-Treated Young Mice

**Figure 2:**
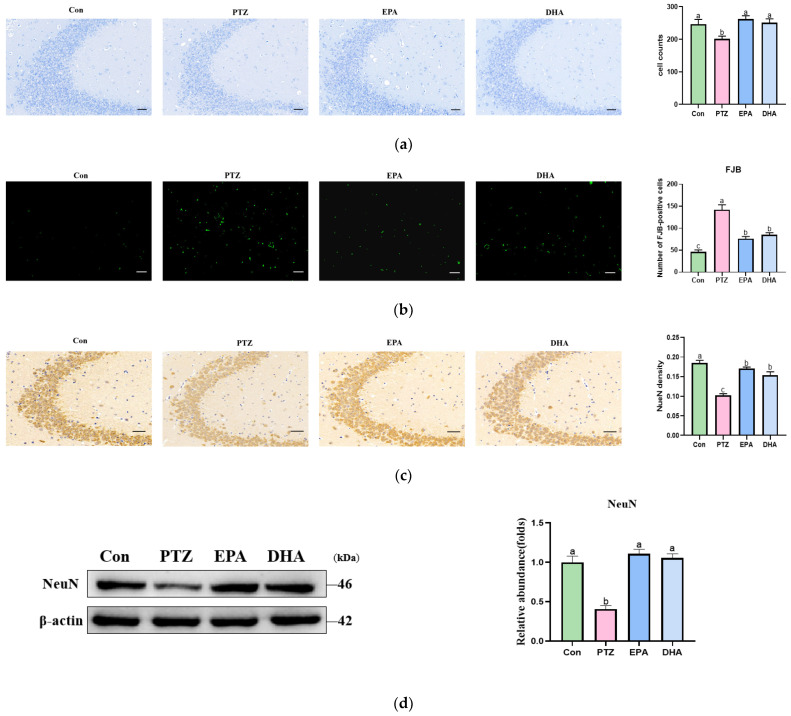
Effects of EPA and DHA on hippocampal neuron injury in PTZ-treated young mice. (**a**) Representative Nissl staining images of the hippocampus; (**b**) Fluoro-Jade B (FJB) staining; (**c**) representative images of NeuN immunohistochemistry staining (*n* = 3), Scale bar, 50 µm; (**d**) representative Western blots and densitometry of NeuN. For quantification, a histogram was generated, where the group with the highest value is designated as a, followed by b–c. Groups with identical marker letters indicate no statistically significant differences, while those with different letters suggest significant differences between each group. The data are presented as mean + SEM (*n* = 3), and differences among groups were evaluated using one-way ANOVA, with significant differences indicated by different letters based on Student’s *t*-test.

Hippocampal neuronal injury was assessed using Nissl staining, which revealed a significant loss of neurons in the CA3 region after PTZ treatment (Figure 2a, *p* < 0.05). In contract to the Con group, the PTZ group exhibited evidence of extensive neuronal degeneration (Figure 2b). NeuN was analyzed to identify neuronal damage. Western blot revealed a significant decrease in the expression of NeuN protein in the PTZ group compared to the Con group (Figure 2d, *p* < 0.05). These findings were consistent with the results of the immunohistochemistry (Figure 2c). However, these forms of neuronal damage could be effectively alleviated by administration of EPA and DHA.

### 3.3. Effects of EPA and DHA on Hippocampal Myelin Damage in PTZ-Treated Young Mice

**Figure 3:**
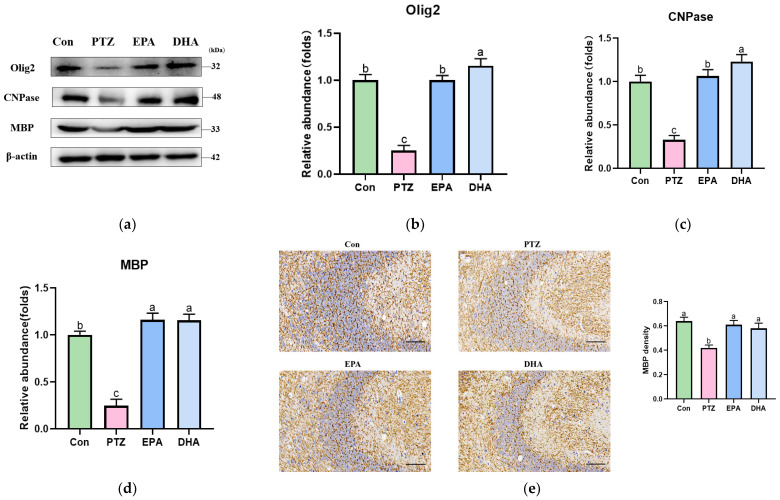
Effects of EPA and DHA on hippocampal myelin damage in PTZ-treated young mice. (**a**) Representative Western blots and (**b**–**d**) densitometry of Olig2, CNPase, and MBP; (**e**) immunohistochemical staining of MBP (*n* = 3), Scale bar, 50 µm. Protein levels are normalized to β-actin, which served as loading control and reproduced with Con group. Values are indicated as the mean + SEM (*n* = 7); *p* < 0.05 was considered to indicate statistically significant. For quantification, a histogram was generated, where the group with the highest value is designated as a, followed by b–c. Groups with identical marker letters indicate no statistically significant differences, while those with different letters suggest significant differences between each group. Differences among groups were evaluated using one-way ANOVA, with significant differences indicated by different letters based on student’s *t*-test.

To further elucidate PTZ-induced impairments in myelin, we analyzed the alterations in oligodendrocyte-related proteins. Western blotting was used to determine the levels of Olig2, CNPase, and MBP, a crucial constituent of myelin. The analysis revealed a significant decrease in Olig2, CNPase, and MBP in the PTZ group compared to that in the Con group (Figure 3a–d). Treatment with DHA and EPA significantly elevated these proteins, and DHA was superior in improving CNPase activity (Figure 3c, *p* < 0.05). Additionally, the immunohistochemistry results for MBP were consistent with those of the Western blotting (Figure 3e, *p* < 0.05).

### 3.4. Effects of EPA and DHA on Hippocampal Microglia Polarization in PTZ-Treated Young Mice

**Figure 4:**
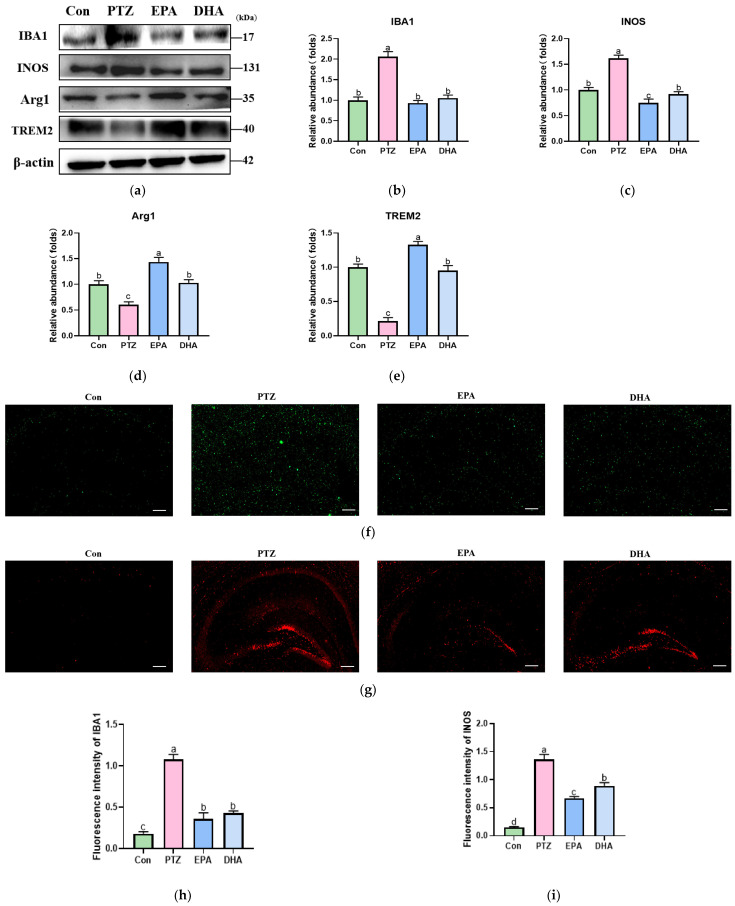
Effects of EPA and DHA on hippocampal microglia polarization in PTZ-treated young mice. (**a**) Representative Western blots and (**b**–**e**) densitometry of IBA1, INOS, Arg1, and TREM2. Protein levels are normalized to β-actin, which served as loading control and reproduced with Con group. Values are indicated as the mean + SEM (*n* = 7). (**f**) Immunofluorescence staining for IBA1 in the hippocampus; (**g**) immunofluorescence staining for INOS in the hippocampus, scale bars, 200 µm, (*n* = 3); (**h**) fluorescence intensity of IBA1 and (**i**) INOS. *p* < 0.05 was considered to indicate statistical significance. For quantification, a histogram was generated, where the group with the highest value is designated as a, followed by b–d. Groups with identical marker letters indicate no statistically significant differences, while those with different letters suggest significant differences between each group. Differences among groups were evaluated using one-way ANOVA, with significant differences indicated by different letters based on Student’s *t*-test.

To investigate the impact of DHA and EPA on microglial activation and polarization, we performed Western blotting to detect markers associated with microglial activation and polarization. Compared to the Con group, the PTZ group demonstrated a significant upregulation of IBA1 (activation) and INOS (M1 polarization) while exhibiting a downregulation of Arg1 (M2 polarization) (Figure 4a–d, *p* < 0.05). We also analyzed the alterations in TREM2, which is known to regulate microglial M1/M2 polarization. Similarly, a significant decrease in TREM2 expression was observed in the PTZ group compared to that in the Con group (Figure 4e, *p* < 0.05). The administration of EPA and DHA reversed these effects. Interestingly, EPA showed a more pronounced enhancement of Arg1 and TREM2 compared to DHA (Figure 4d,e, *p* < 0.05).

### 3.5. Effects of EPA and DHA on NLRP3 Inflammasome Activation in PTZ-Treated Young Mice

**Figure 5:**
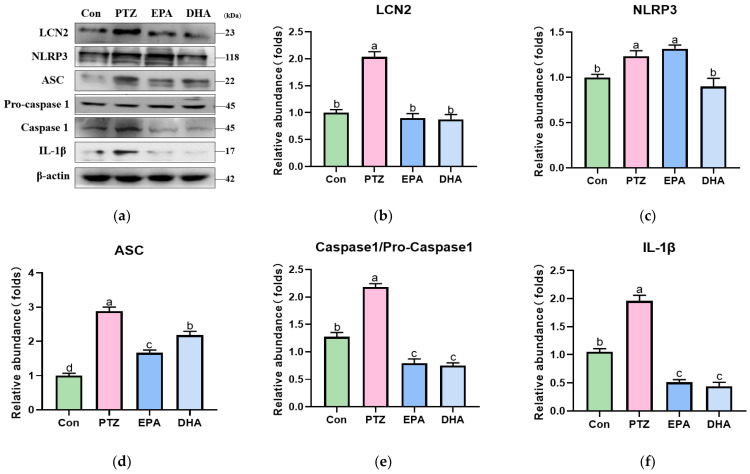
Effects of EPA and DHA on NLRP3 inflammasome activation in PTZ-treated young mice. (**a**) Representative Western blots and (**b**–**f**) densitometry of LCN2, NLRP3, ASC, Caspase1/pro-Caspase1, and IL-1β. Protein levels are normalized to β-actin, which served as loading control and reproduced with Con group. Values are indicated as the mean + SEM (*n* = 7). *p* < 0.05 was considered to indicate statistical significance. For quantification, a histogram was generated, where the group with the highest value is designated as a, followed by b–d. Groups with identical marker letters indicate no statistically significant differences, while those with different letters suggest significant differences between each group. Differences among groups were evaluated using one-way ANOVA, with significant differences indicated by different letters based on Student’s *t*-test.

To investigate the involvement of the NLRP3 inflammasome in PTZ-induced depression in young mice, and to confirm the impact of DHA and EPA on its activation, the inflammasome NLRP3 was examined by Western blotting. Compared to the Con group, the PTZ group exhibited a significant increase in the protein expression levels of NLRP3, ASC, Caspase-1/pro-Caspase-1, and IL-1β (Figure 5, *p* < 0.05), indicating the activation of NLRP3 inflammasome. However, NLRP3 inflammasome activation is inhibited by dietary supplementation with EPA or DHA. In contrast to DHA, EPA exhibited decreased ASC levels and a lower IL-1β/pro-IL-1β ratio, while NLRP3 remained unchanged. Increased LCN2 levels in the brain tissue can induce neuroinflammation via NLRP3 inflammasome activation [41]. Our results revealed that the protein expression levels of LCN2 were significantly elevated in the PTZ group (Figure 5b). However, the administration of EPA and DHA decreased the protein expression of LCN2.

### 3.6. Effects of EPA and DHA on NOX2-Mitochondrial Oxidative Stress in PTZ-Treated Young Mice

**Figure 6:**
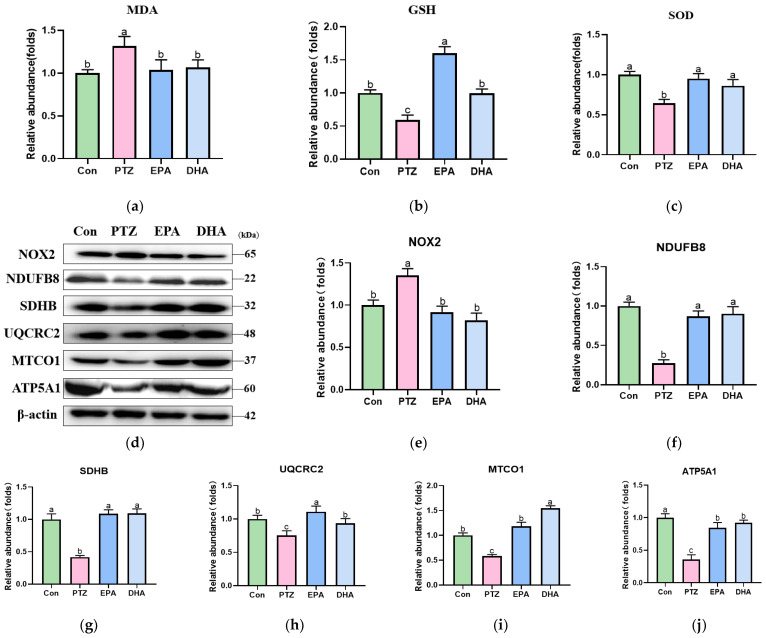
Effects of EPA and DHA on Nox2-mitochondrial mediated oxidative stress in PTZ-treated young mice. (**a**) The level of MDA; (**b**) GSH; (**c**) the activity of SOD; (**d**) representative Western blots, and (**e**–**j**) densitometry of NDUFB8, SDHB, UQCRC2, MTCO1, and ATP5A1. Protein levels are normalized to β-actin, which served as loading control and reproduced with Con group. Values are indicated as the mean + SEM (*n* = 7), and *p* < 0.05 was considered to indicate statistical significance. For quantification, a histogram is generated where the group with the highest value is designated as a, followed by b–c. Groups with identical marker letters indicate no statistically significant differences, while those with different letters suggest significant differences between each group. Differences among groups were evaluated using one-way ANOVA, with significant differences indicated by different letters based on Student’s *t*-test.

NLRP3 inflammasome activation has been demonstrated to promote the activation of oxidative stress [19]. Therefore, we studied the effects of DHA and EPA on oxidative stress in PTZ-treated young mice. In comparison to the Con group, PTZ treatment significantly elevated the levels of MDA and reduced GSH levels and SOD activity in the hippocampus of mice (Figure 6a–c, *p* < 0.05), indicating that PTZ induced oxidative stress. Oxidative stress is characterized by the excessive production of ROS and damage to the mitochondrial electron transport chain (ETC). Based on the premise that mitochondrial ETC and NOX2 are the primary sources of ROS [42,43], the protein levels of key enzymes in the mitochondrial ETC (including Complex I (NDUFB8), Complex II (SDHB), Complex III (UQCRC2), Complex IV (MTCO1), and Complex V (ATP5A1)) and NOX2 were analyzed. In the PTZ group, the expression of NDUFB8, SDHB, UQCRC2, MTCO1, and ATP5A1 was significantly downregulated, whereas NOX2 expression was upregulated compared to that in the Con group (Figure 6d–j, *p* < 0.05). In contrast, the administration of EPA or DHA significantly reversed these alterations (*p* < 0.05). Notably, the EPA prioritized UQCRC2, whereas DHA demonstrated an advantage over MTCO1.

## 4. Discussion

Depression is the most common comorbid condition in childhood epilepsy, leading to a poor quality of life and a worse prognosis for seizure control. However, no effective therapy is available for this disease, and the mechanism by which it develops remains unclear. In this study, we investigated the effects of EPA and DHA on epileptic depression in young PTZ-treated mice. Administration of either EPA or DHA effectively alleviated PTZ-induced epileptic seizures and depressive symptoms in a young mouse model, with EPA demonstrating greater efficacy in improving depression. Further analyses revealed that supplementation with EPA and DHA effectively mitigated neuronal degeneration, myelin damage, oxidative stress, and neuroinflammation.

Studies have established that myelin damage is common in both epilepsy and depression, suggesting that demyelination may serve as a bridge between epilepsy and depression [44,45,46]. In our recent study, we observed a significant decrease in the protein expression levels of MBP, CNPase, and Olig2 in the hippocampus of young mice treated with PTZ. Dietary intake of EPA or DHA significantly elevated the expression of these proteins, indicating that both EPA and DHA possess the ability to alleviate PTZ-induced myelin damage by facilitating the differentiation and maturation of oligodendrocytes in the hippocampus of young mice. Therefore, we hypothesized that the positive effects of EPA and DHA on myelin could serve as a significant cellular foundation for the alleviation of epileptic depression.

Microglia, the principal innate immune cells of the central nervous system, play a crucial role in maintaining normal brain function. Microglia are rapidly activated and polarized to the M1/M2 phenotype in response to their microenvironment [12]. Accumulating evidence suggests that an imbalanced polarization of M1/M2 microglia is associated with various neurological disorders, including depression and epilepsy [10,47]. Inhibiting M1 and/or inducing the M2 phenotype have been proposed as effective therapeutic strategies for epilepsy [48] and depression [49]. The triggering receptor expressed on myeloid cell-2 (TREM2) is highly expressed in microglia and plays a crucial role in regulating microglial polarization and neuroinflammation [50]. Suppression of TREM2 triggers microglial activation and a proinflammatory phenotype, resulting in depression-like behavior [51].

In the PTZ-treated model, we observed a significant decrease in iNOS with concurrent increases in TREM2 and Arg1 following dietary supplementation with DHA and EPA. These findings suggested that the administration of either EPA or DHA effectively inhibited microglial overactivation and promoted microglial M2 polarization in PTZ-treated young mice. Notably, the EPA group exhibited higher protein levels of TREM2 and Arg1 than the DHA group, suggesting that EPA promotes TREM2-mediated microglial M2 polarization. This may have partly contributed to the superior effectiveness of EPA in alleviating depression in PTZ-treated mice. The NLRP3 inflammasome has been recognized as a significant contributor to neuroinflammation by activating caspase-1, which leads to the production of inflammatory factors such as IL-1β [52]. Researchers have previously shown a critical role of the NLRP3 inflammasome in the pathogenesis of depression [53,54], and there is an association between the inhibition of NLRP3 inflammasome assembly and the amelioration of depression-like behaviors in animal models [25,55]. LCN2, a member of the highly heterogeneous lipocalin family of secretory proteins, is released by injured neurons that activate microglia and amplify their M1 polarization [56,57]. LCN2 activates the NLRP3 inflammasome by directly upregulating the NLRP3 inflammasome complex in LPS-treated macrophages [58]. LCN2 deficiency diminishes NLRP3 inflammasome activation and IL-1β production in spinal-cord-injured mice with SCI [59]. Our current findings consistently demonstrate increased expression of LCN2, NLRP3 inflammasome complex proteins, and IL-1β in PTZ-treated young mice. These results suggest that the LCN2-NLRP3 inflammasome pathway is involved in PTZ-induced depression in young mice. However, supplementation with EPA and DHA comparably reduced the levels of LCN2 protein and NLRP3 inflammasome complex proteins, resulting in decreased IL-1β production. Interestingly, unchanged NLRP3 levels and lower ASC levels were observed in the EPA group. This suggests that the inhibition of the NLRP3 inflammasome by DHA and EPA relies on LCN2 but in a different way.

A growing body of evidence indicates that inflammation induces oxidative stress, and that oxidative stress promotes inflammation [43]. Oxidative stress is believed to be the leading mechanism underlying depression and epilepsy [60]. Consistently with previous studies [35,61], our data revealed an increase in MDA, a marker of lipid peroxidation, and decreases in GSH and SOD, indicating the occurrence of oxidative stress in the hippocampus of PTZ-treated young mice. Numerous studies have demonstrated a strong association between mitochondrial dysfunction and the development of neurological disorders, such as major depressive disorder and epilepsy [42,62,63]. NOX, an enzyme complex, facilitates the generation of cellular ROS by transferring an electron from NADPH to oxygen [64]. Hu et al. [43] reported that NOX2 induced mitochondrial ROS production. Moreover, inhibition of NOX2 can alleviate oxidative stress and anxiety- and depression-like behaviors in PTZ-treated mice [65]. Our findings revealed that the administration of EPA and DHA reduced NOX2 and upregulated proteins related to mitochondrial metabolism (complexes I-V). Interestingly, the EPA group showed higher levels of complex III core subunit (UQCRC2), whereas the DHA group exhibited higher levels of complex IV core subunit (MTCO1) protein. Therefore, we conclude that EPA and DHA exert beneficial effects on PTZ-induced depression by inhibiting NOX2-mitochondrial oxidative stress, albeit in slightly different ways.

DHA is the most abundant ω-3 fatty acid in the brain and plays a crucial role in brain development. In contrast, as a DHA precursor, EPA accumulates to a lesser extent in the brain. Our previous study found that administration of EPA or DHA comparably increased brain DHA levels, whereas only mice treated with EPA exhibited a significant increase in cerebral EPA. Several clinical studies have indicated that EPA administration significantly improves depression and has a greater antidepressant effect than DHA in patients with depressive disorders [48,49,50]. A previous meta-analysis reported that EPA (purity > 60%) has significantly greater efficacy than DHA in treating depression [66]. Our findings provide further evidence, supporting the superior efficacy of EPA over DHA in improving PTZ-induced depression in young mice. Furthermore, purified EPA was more effective than DHA in reversing patients’ proinflammatory profile. In line with this, our results demonstrated that EPA exhibited a stronger preference in reducing the M1/M2 polarization ratio in activated microglia. The brain mainly contains arachidonic acid (AA), which is an ω-6 fatty acid. It is abundant in the membrane phospholipids of inflammatory cells and produces proinflammatory oxylipins [67]. The accumulation of AA during epileptic seizures can damage cell membranes and mitochondria through lipid peroxidation [68,69]. In the present study, supplementation with EPA and DHA resulted in a reduction in the brain—AA ratio, leading to a decrease in AA oxylipins [67,70] and an increase in DHA/EPA-derived oxylipins possessing neuroinflammation-inhibitory properties [66,71]. In fact, the active metabolites of EPA and DHA (i.e., oxylipins) differ, which may contribute to their differential efficiency and anti-inflammatory mechanisms. Oxylipins from EPA and DHA have unique anti-inflammatory and pro-resolution properties that may be useful for developing novel depression management strategies.

## 5. Conclusions

In conclusion, our findings suggest that dietary DHA and EPA may protect against epileptic depression by improving myelin damage, inhibiting neuroinflammation by reducing mitochondrial metabolic impairment, and promoting microglial M2 polarization in a young PTZ-treated mouse model. Notably, EPA demonstrated superior efficacy compared to DHA. These findings suggest that dietary interventions involving EPA or DHA may be effective, with EPA being the preferred choice.

## Data Availability

Data are contained within the article and Supplementary Materials.

## Supplementary Materials

The following supporting information can be downloaded at: https://www.mdpi.com/article/10.3390/antiox12122079/s1. Table S1: Ingredient and main fatty acid compositions of experimental diets.
