# Supplementary material for: DHA and EPA Alleviate Epileptic Depression in PTZ-Treated Young Mice Model by Inhibiting Neuroinflammation through Regulating Microglial M2 Polarization and Improving Mitochondrial Metabolism
Source: Antioxidants (Basel). 2023 Dec 6;12(12):2079. doi: 10.3390/antiox12122079 (PMC10740521; doi:10.3390/antiox12122079)
Supplement: Supplementary file 1 [file antioxidants-12-02079-s001.zip › antioxidants-2715224-supplementary.pdf]

Table S1. Ingredient and main fatty acid compositions of experimental diets

**Ingredient(g/kg)**	**AIN93G**	**EPA**	**DHA**
Corn starch	397.5	397.5	397.5
Dextrin	132	132	132
Sucrose	100	100	100
Casein	200	200	200
Powdered cellulose	50	50	50
Soybean oil	70	60	60
Mineral mix	35	35	35
Vitamin mix	10	10	10
Choline bitartrate	2.5	2.5	2.5
L-Cystine	3	3	3
EPA ethyl ester	-	10	-
DHA ethyl ester	-	-	10
t- buthlhydroquinone	0.014	0.014	0.014
Fatty acids composition (%)			
C16 :0	11.76	9.6	10. 18
C18 :0	4.06	3.3	3.61
C18 :1	23.9	19.4	20.3
C18 :2n-6	48.68	38.0	40.25
C18 :3n-3	5 72	4 5	4 79
C20 :4n-6	-	1.1	-
C20 :5	-	17.0	-
C22 :6	-	2.2	17.31

Note :"-" , none detected. aModified AIN-93G.
